# Head and neck cancer care: advanced molecular and genetic diagnostics

**DOI:** 10.17179/excli2024-7727

**Published:** 2024-10-10

**Authors:** Rabab Fatima

**Affiliations:** 1 Department of Chemistry, University of Petroleum and Energy Studies (UPES), Energy Acres, Dehradun 248007, India

## ⁯⁯

Head and neck cancers (HNCs) include neoplasms of the upper aero digestive tract (UADT) such as nasal cavity, oral cavity, paranasal sinuses, larynx, pharynx, and salivary glands. They represent the seventh most commonly occurring malignancy accounting for 6 % of solid tumors. Epidemiological data play a crucial role in healthcare planning, their scarcity in certain regions hinders efficient resource distribution and the development of effective health policies. Substantial progress has been made in understanding the molecular mechanisms and progression of HNC through surgery, radiation, chemotherapy, and immunotherapies. Oral metronomic chemotherapy offers significant potential for palliative care and managing recurrent and/or head and neck squamous cell carcinoma (R/M HNSCC). However, its role in adjuvant therapy particularly in maintenance and neoadjuvant settings remains under-evaluated with limited evidence. Despite these advancements, challenges such as tumor heterogeneity, drug toxicity, recurrence, and resistance continue to hinder optimal outcomes. Additionally, the need for specific imaging modalities for different anatomical areas can lead to inconsistencies in diagnosis and staging, particularly with PET/CT. The observed discrepancies between clinical trial results and real-world clinical practice highlight a gap in effectively translating research findings. 

The emergence of targeted next-generation sequencing of a curated 50 gene panel reported single-nucleotide polymorphisms (SNPs) in HPV-positive and HPV-negative HNSCC cases. SNPs in tumor suppressor genes were more prevalent in HPV-negative cases deciphering divergent molecular pathogenesis and genetic dichotomy. Long-read sequencing technologies such as Oxford Nanopore and PacBio sequencing have enabled the detection of complex structural variants, distinct ionic signatures and longer nucleotides sequence and fusion genes such as FGFR3 in HPV-negative oropharyngeal cancers as compared to traditional sequencing. Single-cell DNA and RNA sequencing technologies are being refined for broader application, to specifically enhance variant calling in low-cellularity samples. Puram et al. characterized intratumoral heterogeneity in oral cavity squamous cell carcinoma, where distinct subpopulations with varying degrees of epithelial-mesenchymal transition potentially correlated with the metastatic potential. From 6,000 transcriptomes of 18 HNSCC patients, they established p-EMT as an independent marker predicting nodal metastasis, tumor grade, and adverse pathological features (Puram et al., 2017[9]). Liquid biopsy provides comprehensive genetic profiling through circulating tumor DNA (ctDNA) and exosomes, potentially capturing tumor heterogeneity more effectively than single-site tissue biopsies and sequencing. The LIONESS study (Liquid BIOpsy for MiNimal RESidual DiSease Detection in Head and Neck Squamous Cell Carcinoma) performed personalized ctDNA profiling at a molecular level, aiming to improve early detection of residual tumor cells (Flach et al., 2022[5]). A critical component of ongoing FOCUS study (NCT05075122) using hTERT-targeted cancer vaccine UV1 in combination with Pembrolizumab, explored serial liquid biopsy to predict disease progression in R/M HNSCC (Brandt et al., 2024[1]). The study identified potentially emerging resistant tumor subclones from previously associated genes reported for resistance to checkpoint inhibitors. 

The presence of recurrent mutations in TP53, CDKNA2, NOTCH 1, PTEN, PIK3CA, and HRAS is responsible for significant inter-tumor heterogeneity in HNSCC. Genome-wide screens utilizing microarray and next-generation sequencing technologies detect EGFR mutations and aid in proper use of EGFR inhibitors such as Cetuximab. With the recent endorsement of checkpoint inhibitors for oral squamous cell carcinoma (OSCC), genetic profiles have identified immune-active and exhausted subtypes, providing insights into patient immune status and inform the development of novel immunotherapies (Chai et al., 2020[2]). The CheckMate 141 and KEYNOTE-048 trials presently serve as illustrative, practice-changing landmarks that have immune checkpoint inhibitors (ICIs) inclusion in the treatment guidelines for HNSCC (Voortman, 2024[10]). Genome editing technologies leveraging DNA-dependent polymerases (DDPs) offer several benefits for implementing diverse genetic changes. The click editing platform, which integrates DDPs with RNA-programmable nickases can effectively install a diverse array of edits in neoplasms (da Silva et al., 2024[3]). Spatial transcriptomics via 10x Genomics Visium elucidated crucial insights into the tumor architectures revealing HPV-specific cellular compositions, PKM2 for cancer stemness and EPHA2 pathway for angiogenesis in HPV-negative cases while ectopic lymphoid structures were found in HPV-positive samples (Lee et al., 2024[6]).

AI-powered analysis of intratumoral tumor infiltrating lymphocyte density highlighted a robust correlation with favorable responses to ICI's in R/M HNSCC. Peng et al. in this regard, developed a deep learning model for automated detection and grading of oral epithelial dysplasia using 4 convolutional neural networks from 56 oral leukoplakia slides projecting the accuracy of E MOD plus (Peng et al., 2024[8]). Recent technological advancements, including CRISPR-Cas9 screening for high-throughput gene identification, patient-derived xenografts for preserving tumor heterogeneity, and patient-derived organoids for mimicking native tissue architecture, have propelled our understanding of drug resistance and the identification of novel predictive targets. Patient-derived HNSCC organoids have emerged as a valuable 3D model for evaluating EGFR-targeted photodynamic therapy (PDT) accurately reflecting varying EGFR expression levels correlating to PDT responses (Driehuis et al., 2019[4]). A strong rationale for targeting MINK1 through AKT/MDM2/p53 axis to overcome chemoresistance in OSCC, for patients with 5 FU resistant tumors was performed using CRISPR/Cas9 based kinome knockout screening (Mohanty et al., 2022[7]). The identification of Lestaurtinib as a MINK1 inhibitor further offered a promising approach for translating these findings into clinical applications significantly reducing tumor burden.

The advancements discussed here represent a significant leap forward in personalized HNC management, offering the potential for more accurate diagnosis, refined prognostic stratification, and tailored treatment strategies. Close collaboration between clinicians and researchers will soon allow for realization of these advanced molecular and genetic tools for unprecedented precision and transformative care in head and neck neoplasms.

## Declaration

### Funding

The study received no funding.

### Conflict of interest

The author declares no conflict of interest.
